# Genetic pleiotropy in complex traits and diseases: implications for genomic medicine

**DOI:** 10.1186/s13073-016-0332-x

**Published:** 2016-07-19

**Authors:** Jacob Gratten, Peter M. Visscher

**Affiliations:** Queensland Brain Institute, The University of Queensland, Brisbane, QLD 4072 Australia

## Editorial summary

Several recent papers have used summary results from genome-wide association studies to characterize genetic overlap between human complex traits and common diseases. The emerging evidence is that individual DNA variants frequently influence multiple phenotypes, often in unexpected ways. This has important implications for genomic medicine and for the application of genome editing.

## Pervasive pleiotropy

Pleiotropy is the phenomenon in genetics whereby a DNA variant influences multiple traits. We have known for decades that pleiotropy is widespread because in plant and animal breeding, and in laboratory selection experiments, when selection is applied to one trait, the mean of other traits also changes from generation to generation. The response to selection reflects the genetic correlation between traits, which summarizes the genome-wide average effects of pleiotropy at shared loci. In studies of human traits, estimates of the genetic correlation can be obtained using traditional family-based study designs [1], or high-dimensional genetic data from genome-wide association studies (GWAS) [2, 3]. These estimates provide no information on where in the genome DNA variants with pleiotropic effects exist, on whether individual shared variants have concordant or discordant effects across traits, or if the effects are causally related as opposed to operating through independent biological pathways. Several new papers have described methods that address these questions using GWAS summary data [4–6]. Here, we review key advances from these papers that enable more in-depth investigations of pleiotropy, and we discuss their implications for genomic medicine.

## Understanding pleiotropy using GWAS

GWAS have been applied to hundreds of complex traits and common diseases, yielding thousands of genetic associations that surpass accepted standards for statistical significance [7]. Several studies have used results from GWAS to systematically identify genetic variants associated with multiple traits, both across the breadth of human biomedical traits and disorders [5, 8] and for groups of related diseases with prior evidence for a shared etiology (for example, immune-mediated diseases [9]). As expected, pleiotropy is commonly found for variants associated with traits in the same “domain”—for example, Parkes and colleagues [9] identified 71 genome-wide significant variants associated with two or more of six immune-mediated diseases—but there are interesting subtleties to this genetic overlap. For instance, although many shared variants have correlated and concordant effects, a surprising number are discordant, insomuch as they increase risk for one disorder (such as ankylosing spondylitis) but are protective for another (such as rheumatoid arthritis [9]). Conversely, other studies have revealed unexpected associations between traits previously thought to be biologically unrelated. For example, in an analysis of GWAS summary data for 42 traits, Pickrell and coworkers [5] reported the identification of a variant (from among a total of >300 pleiotropic loci) in the *ABO* gene, which determines blood group, that was associated with both coronary artery disease (CAD) and tonsillectomy (among other traits). A major strength of this approach is that pleiotropy can be investigated without the need to measure phenotypes in the same individuals, meaning that confounding by environmental factors is unlikely.

A genetic correlation between traits or diseases can arise due to pleiotropy, as described above, or because of heterogeneity, which refers to the situation in which a proportion of cases for one disease have been misclassified as another. Han and colleagues [4] recently proposed a method, termed breaking up heterogeneous mixture based on cross-locus correlations (BUHMBOX), for distinguishing between these possibilities. In order to detect heterogeneity involving the misdiagnosis of disease B cases as disease A, the approach tests for an excess of positive correlations between independent disease B risk alleles in individuals with disease A—something that is not expected under pleiotropy. Using this approach, which requires GWAS summary data for individuals with disease B and genotype data for individuals with disease A, the authors reported evidence for heterogeneity between seronegative and seropositive forms of rheumatoid arthritis, presumably due to misclassification of a subset of seropositive cases [4]. Heterogeneity is likely to be widespread in complex traits and common diseases, and may be one explanation for the dearth of genetic associations identified for psychiatric disorders such as major depression. BUHMBOX offers a promising tool to differentiate pleiotropy from heterogeneity, although a caveat is that statistical power is limited when the proportion of heterogeneity is low, and yet high levels of heterogeneity may be more likely if pleiotropy is extensive.

Pleiotropy can involve a genetic variant having effects on two or more traits via independent biological pathways, for instance due to effects in different tissues, or because the effect of the variant on one trait is causally related to variation in another trait. Pickrell and colleagues [5] recently put forward an innovative method to tease apart these possibilities, by testing if variants associated with an increase in one trait are always associated with a proportional increase in the other trait, but not the other way around. Using this approach they confirmed the widely accepted causal relationship between low-density lipoprotein cholesterol and CAD, and identified several other plausible causative relationships, including between body mass index (BMI) and both triglyceride level and risk of type 2 diabetes (that is, BMI-increasing alleles have correlated effects on triglycerides and type 2 diabetes risk, but not vice versa). These are exciting developments because they imply that causal relationships can be uncovered more cheaply and rapidly through statistical analysis of genetic data than by performing randomized controlled trials. However, as the authors note, caution is needed in interpretation because the observed phenotype, which is presumed to be causal, may in fact be genetically correlated with another, unobserved phenotype that is the true causal factor.

A form of pleiotropy commonly encountered in GWAS is that trait- or disease-associated single nucleotide polymorphisms (SNPs) are frequently also associated with variation in gene expression (expression quantitative trait loci (eQTLs)) and/or DNA methylation (methylation quantitative trait loci (meQTLs)). Recently, Zhu and coworkers [6] proposed a novel method termed summary-data-based Mendelian randomization (SMR) for combining GWAS summary data with eQTL and meQTL data in order to isolate the most likely functional gene or regulatory element underlying statistical associations for complex traits and common diseases. They also proposed a method (heterogeneity in dependent instruments (HEIDI)) that can distinguish pleiotropy from linkage, since the observation that a trait- or disease-associated SNP is also a *cis*-eQTL may actually be due to linkage disequilibrium between the sentinel SNP and other SNPs that are independently causally related to gene expression and the trait or disease under investigation.

We have emphasized evidence for pleiotropy from GWAS here, but pleiotropy is also evident for rare mutations underlying Mendelian disorders. Indeed, specific “syndromes” can be diagnosed on the basis of the combination of phenotypes that arise from the same causal mutation. For example, Rett syndrome, caused by mutations in the *MECP2* gene, which encodes a protein important for nerve cell function, is a neurological disorder characterized by intellectual disability and apraxia that frequently presents with short stature and gastrointestinal problems. Another example of a mutation with phenotypic effects spanning different biological “domains” is the cystic fibrosis transmembrane conductance regulator gene (*CFTR*) ΔF508 mutation causing cystic fibrosis, a disease of the lung that is also associated with male infertility.

## Implications for genomic medicine

Pervasive pleiotropy has important implications for genomic medicine, particularly as we move into the era of personalized medicine and genome editing. One issue is that focusing on the effect of a mutation or polymorphism on a single disease may be inadequate, since specific genetic variants may show strong associations with multiple traits but in opposite directions [9]. This is especially salient in the context of identifying molecular targets for drug development [8], and when contemplating “fixing” mutations using genome editing approaches such as the CRISPR-Cas system, since this might have unexpected genetic, and therefore phenotypic, side effects. We find more evidence for pleiotropy the more we look, and yet the vast majority of phenotypes are never measured. Indeed, one could ask, given the enormous dimensionality of the phenome, how likely it is that functional variants exist without pleiotropic effects. Herein lies a major challenge for the field, as the possibility of detrimental effects (for example, as a consequence of genome editing) may be hard to rule out.

To some extent, this problem will be ameliorated by the availability of GWAS data from very large studies, such as the UK Biobank and US National Institutes of Health Precision Medicine Initiative, in which participants are measured for a large number of phenotypes. In parallel, large-scale genome sequencing studies matching data on rare mutations with deep phenotyping (for example, [10]) will help to characterize the phenotypic spectrum of gene-disrupting mutations in specific genes, and thus clarify if such events are associated solely with deleterious phenotypic outcomes as opposed to a mix of detrimental and beneficial consequences. We can expect these studies to deliver many new and unexpected discoveries on genome–phenome associations, including plausible causal trait relationships. We anticipate that pleiotropy will come to be recognized as a (near) universal property of genetic variants contributing to human phenotypic variation. The limiting factor in progress towards a more complete understanding of the relationship between genome and phenome will be the availability of high-dimensional phenotype data.

## Abbreviations

BMI, body mass index; BUHMBOX, breaking up heterogeneous mixture based on cross-locus correlations; CAD, coronary artery disease; eQTLs, expression quantitative trait loci; GWAS, genome-wide association studies; HEIDI, heterogeneity in dependent instruments; meQTLs, methylation quantitative trait loci; SMR, summary-data-based Mendelian randomization; SNP, single nucleotide polymorphism.
